# A framework for the first-person internal sensation of visual perception in mammals and a comparable circuitry for olfactory perception in *Drosophila*

**DOI:** 10.1186/s40064-015-1568-4

**Published:** 2015-12-30

**Authors:** Kunjumon I. Vadakkan

**Affiliations:** Division of Neurology, Department of Medicine, University of Toronto, Sunnybrook health Sciences Centre, 2075 Bayview Ave. Room A4-08, Toronto, ON M4N3M5 Canada; Neurosearch Center, 76 Henry St., Toronto, ON M5T1X2 Canada

**Keywords:** Perception, Visual perception, Olfactory perception, Semblance hypothesis, Inter-postsynaptic functional LINK, Cortical columns, Internal sensation, First-person frame of reference, First-person internal sensation, Flicker fusion frequency, *Drosophila* olfaction

## Abstract

Perception is a first-person internal sensation induced within the nervous system at the time of arrival of sensory stimuli from objects in the environment. Lack of access to the first-person properties has limited viewing perception as an emergent property and it is currently being studied using third-person observed findings from various levels. One feasible approach to understand its mechanism is to build a hypothesis for the specific conditions and required circuit features of the nodal points where the mechanistic operation of perception take place for one type of sensation in one species and to verify it for the presence of comparable circuit properties for perceiving a different sensation in a different species. The present work explains visual perception in mammalian nervous system from a first-person frame of reference and provides explanations for the homogeneity of perception of visual stimuli above flicker fusion frequency, the perception of objects at locations different from their actual position, the smooth pursuit and saccadic eye movements, the perception of object borders, and perception of pressure phosphenes. Using results from temporal resolution studies and the known details of visual cortical circuitry, explanations are provided for (a) the perception of rapidly changing visual stimuli, (b) how the perception of objects occurs in the correct orientation even though, according to the third-person view, activity from the visual stimulus reaches the cortices in an inverted manner and (c) the functional significance of well-conserved columnar organization of the visual cortex. A comparable circuitry detected in a different nervous system in a remote species—the olfactory circuitry of the fruit fly *Drosophila melanogaster*—provides an opportunity to explore circuit functions using genetic manipulations, which, along with high-resolution microscopic techniques and lipid membrane interaction studies, will be able to verify the structure–function details of the presented mechanism of perception.

## Background

Helmholtz proposed that visual perception is mediated by unconscious inferences using cognitive resources (Helmholtz 1866; Barlow 1990). Even though Helmholtz’s proposal suggested an inseparable nature of perception and cognition (Hatfield 2002), the idea that they are entirely separate processes emerged later (Fodor 1983; Rock 1983). In an alternate approach, a computational hypothesis using the methods of calculus was developed (Marr and Poggio 1979). According to this view, sudden intensity changes at the edges were proposed to correspond with the maximum or minimum of the image intensity values at the zero-crossings in the second derivate. Further work led to the information processing proposal with different stages of operation (Marr 1982). These include two-dimensional (2-D) images falling on to the retina followed by the formation of an intermediate 2.5-D sketch of a viewer-centered description of orientation, contour depth and other properties of visible structures and finally to a 3-D percept. However, cellular mechanisms and experimental verification of these are lacking. Perception was also viewed in terms of variational free-energy minimization based on the assumption that biological systems maximize the Bayesian evidence for their model of the world through an active sampling of sensory information (Friston et al. 2006).

Studies showed that activity in a population of neurons automatically represent probability distributions—namely, probabilistic population codes, over the visual stimulus by using Bayesian rules (Knill and Pouget 2004; Yuille and Kersten 2006). Bayes’s rule was used to calculate edge probability at a given location or orientation in an image based on the surrounding filter population (Ramachandra and Mel 2014). Examinations of how populations of neurons correlate with perceptual spaces across multiple sensory modalities are currently being undertaken (Zaidi et al. 2013) with the preliminary conclusions that (1) the observed invariant representations may be contained within the statistics of distributions of stimuli, and (2) the representation of the finer aspects of the detailed properties utilize temporal neuronal spiking patterns. In another approach, neuronal firing models were proposed to show how the lateral geniculate nucleus (LGN) and the visual cortical areas V1, V2 and V4 utilize visual information to produce 3-D surface percepts (Grossberg and Howe 2003). Models that assume that the neurons compute with probability distributions have examined various aspects of perception (reviews Ballard et al. 2002; McClelland 2013).

All the above approaches have the implicit expectation that certain emergent properties are responsible for the internal sensation of perception similar to other higher brain functions. The necessity to undertake new approaches towards exploring neural networks has been discussed (Tsodyks and Gilbert 2004). However, it is not known at what cellular level such emergence occurs or how the emerging virtual internal sensations can be studied empirically. Studying neurons for nearly a 100 years and neural networks for more than 50 years has not resulted in an understanding of the mechanism for emergence. This has led to the recent initiation of projects examining the anatomical connections (Oh et al. 2014), undertaking electrophysiological simulations (Lasserre et al. 2012) and mapping the activity of sets of neurons in complex brain circuits, followed by the manipulation of their activity to understand the system (Alivisatos et al. 2012). Even though several viewpoints urging the necessity to initiate novel approaches and investigations have been made (Abbott 2008; Edelman 2012; Gallistel and Balsam 2014; Grillner 2014; Laughlin 2014; Marder 2015), such attempts require concepts that deviate substantially from the current ones. An ideal investigative approach should be searching for mechanisms that can explain various findings at multiple levels, which will then allow gold standard method of replicating the mechanism in engineering systems. Undertaking such an approach will require explaining the nodal points within the neuronal circuitry at which various internal sensations emerge, details of the specific mechanisms and the required specific conditions. The present work has gathered motivation from the above and examined the nervous system.

Almost all the current studies examine the third-person observed firing of neurons in the sensory cortices, cellular and biochemical changes, changes in the patterns of utilization of various molecules using imaging studies, and changes in the surrogate markers such as speech and behavior to correlate with the first-person internal sensation of perception. For the systems property of perception that “observes the world from within the system”, the present work examines a mechanism that can provide an internal sensory process for perception by examining it from a first-person frame of reference. Understanding the mechanistic aspects of its operation is an essential step for replication in engineered systems. Problems with access to the first-person internal sensations make it impossible to directly study their formation using biological systems. This can be overcome by hypothesizing a feasible mechanism, by searching for the presence of comparable mechanisms for perception of a different sensation in a different species and by keeping a rigorous standard of replication in engineered systems. The present work is a modification of an abstract presented at the Cognitive Neuroscience Society annual meeting (Vadakkan 2012).

## Neuronal firing and internal sensory process

Neuronal firing (somatic spiking) is one of the different spiking activities observed at different neuronal processes. Others include dendritic spikes and axonal spikes (action potentials). The third-person observed neuronal firing is comparable across different excitatory neuronal populations. However, severe variabilities exist in both the number of input connections (postsynapses or postsynaptic terminals or dendritic spines) and the sets of sensory inputs that can trigger a single neuronal firing. The number of inputs of a neuron ranges from one (passive conductance of potentials between the initial orders of neurons of the visual pathway) to many approximately 5600 (as in a monkey’s visual cortex) to 60,000 (as in a monkey’s motor cortex) (Cragg 1967).

The observed firing of a neuron cannot be directly attributed to the induction of first-person internal sensory elements of the various higher brain functions due to the following reasons. (1) Since different sets of dendritic spine inputs (postsynaptic potentials) can lead to the same action potential, neuronal firing is non-specific with regards to its inputs. For example, in a pyramidal neuron with thousands of inputs, the arrival of nearly any set of 40 excitatory postsynaptic potentials (EPSPs) at the axonal hillock can lead to its firing (Palmer et al. 2014). (2) Secondly, depending on the distance that the postsynaptic potentials travel and the diameter of the dendrites, they degrade as they reach the axon hillock where action potentials are triggered (Spruston 2008). Therefore, contributions of these potentials to neuronal firing vary. (3) Thirdly, several postsynaptic potentials contributing to both sub- and supra-threshold activation of a neuron do not contribute to its firing. Therefore, any internal sensory processing that occurs at the level of the synapses of those postsynaptic terminals will not be reflected on the observed neuronal firing. (4) Fourth, the observed effect of a neuronal firing at its axonal terminals (presynaptic terminals) measured in terms of the firing of the neurons to which they synapse also varies widely depending on the background state of latter’s sub-threshold activation. (5) Finally, it can be argued that the evolutionary conservation of postsynaptic potentials induced at farther locations from the soma most likely attributes to certain functions independent of the neuronal firing since these potentials degrade as they propagate towards the axonal hillock. In this context, large potentials recorded from apical tuft areas need a mention. Dendritic spikes are large potentials initially shown by the focal stimulation of dendrites (Regehr et al. 1993; Polsky et al. 2009; Larkum et al. 2009) and contribution of dendritic spikes towards neuronal firing is dependent on the location and distance from the soma. Dendritic NMDA spike is a synchronous activation of 10–50 neighboring glutamatergic synapses triggering a local regenerative potential (Antic et al. 2010). Recent work shows strong evidence for the presence of dendritic spikes in vivo (Palmer et al. 2012; Cichon and Gan 2015; Sheffield and Dombeck 2015). These findings indicate that the functions occurring at the postsynaptic terminals of the apical tuft dendrites of different cortical order neurons that are anchored to the sub-pial region most likely take place independent of the firing of these cortical neurons.

Sensory information arrives at the input level. The evolutionary conservation of all synaptic activity, whether it contributes to the neuronal firing or not, indicates the presence of a well-maintained functional role residing at the level of the postsynaptic membrane activation (Vadakkan 2015b). This leads to the following questions. What third-person observed features can provide information about a process that induces internal sensory meaning to the system as a first-person property? What equivalent internal sensory meaning can an object in the environment impart to the system contributing to the formation of internal sensation of its perception? The best experimental condition that this can be used to isolate the mechanism for induction of internal sensation is associative learning. This calls for the asking of a specific question—Can associative learning events impart certain changes at the location of convergence of associatively learned stimuli such that at a later time when one of the stimuli arrives, the internal sensation of the second stimulus will be induced? Once a basic structure–function mechanism of operational units for internal sensations is derived, then its modifications can be examined for internal sensations of other higher brain functions such as perception.

## Formation of internal sensations

A mechanism for the formation of first-person internal sensations of memories by a process of semblance formation was explained previously (Vadakkan 2007; 2013). Inter-postsynaptic functional LINKs of various half-lives are hypothesized to be responsible for the induction of different types of internal sensations (Vadakkan 2015b). By an extension of the same mechanism, specific conditions and mechanisms for the internal sensations of perception are derived by incorporating unique structural features observed at the primary sensory cortices such as cortical columns and by properties of visual perception such as homogeneity of rapidly arriving frames of percepts. It can be observed from a large number of electron microscopic (EM) imaging studies that the extracellular matrix space between the neuronal processes is minimal in the cortical areas (Harris and Stevens 1989; Burette et al. 2012). This, combined with the previous findings that the average inter-dendritic spine (inter-postsynaptic) distance in pyramidal neurons is greater than the average spine head circumference (Konur et al. 2003), and astrocytic processes surround the synapses only less than half of the synaptic interface among the 57 ± 11 % of synapses at which they are present in stratum radiatum of the hippocampal area CA1 (Ventura and Harris 1999), increases the probability for the dendritic spines (postsynapses or postsynaptic terminals) of different pyramidal neurons to abut each other.

**Fig. 1:**
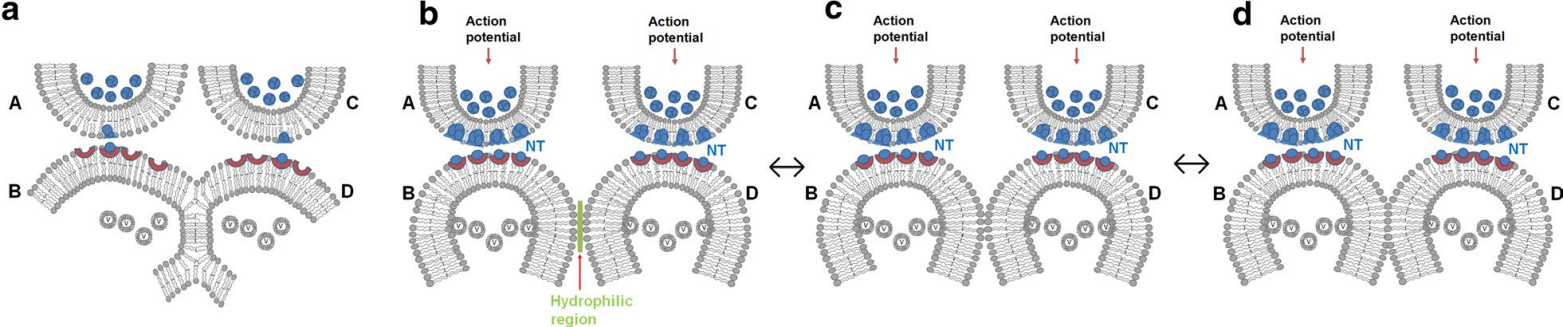
Different possible types of inter-postsynaptic functional LINKs for perception. **a** Innate genetically determined and stabilized hemifused postsynaptic membranes *B* and *D* (*A* and *C* are the presynaptic terminals); **b** two synapses *A*–*B* and *C*–*D* are shown close to each other with their postsynaptic terminals *B* and *D* separated from each other by a hydrophilic region; **c** rapidly reversible direct contact between the postsynaptic membranes *B* and *D* by excluding the inter-membrane hydrophilic medium; **d** rapidly reversible partial inter-postsynaptic membrane hemifusion. Note the partial hemifusion between the postsynaptic membranes *B* and *D*. Since changes associated with perception require to be reversed rapidly back to the ground state at physiological time-scales for continuous perception to occur at the same locations (for perceiving rapidly changing percepts from the environment), the ideal nature of inter-postsynaptic functional LINK is expected to be of the type (**a**) or (**c**). NT: Neurotransmitter (modified from Vadakkan 2015a)

The ability to continuously perceive rapid changes in the environment by the same visual cortical area indicates that the mechanism of perception of rapidly changing environmental stimuli occurs through either pre-existing or rapidly reversible inter-postsynaptic functional LINKs between the dendritic spines of the visual cortical neurons. The lateral spread of activity through these LINKs can contribute to the horizontal component (Vadakkan 2015b) of the observed oscillating potentials at the visual cortex (Herculano-Houzel et al. 1999). Since the formation of inter-postsynaptic functional LINKs is expected to overcome high-energy barriers (Cohen and Melikyan 2004; Martens and McMahon 2008) and is expected to occur at small areas of nearly 10 nm^2^ (Leikin et al. 1987), different types of inter-postsynaptic functional LINKs (Fig. 1) may provide this function. Since repeated mechanisms at the same cortical locations are expected to be used for repeatedly perceiving changing sensory stimuli, two main possibilities can be considered. (1) Innate mechanisms for the formation of stabilized inter-postsynaptic functional LINKs, most likely by the complete inter-postsynaptic membrane hemifusion (Fig. 1a) that are potentially stabilized through the insertion of transmembrane proteins at the hemifused membrane segments. (2) Rapid formation of direct membrane contacts by removing the inter-membrane hydration repulsion (LeNeveu et al. 1976; Rand and Parsegian 1989; Israelachvili and Wennerström 1996; Kanduč et al. 2014) during perception and their rapid reversal is another possible mechanism. Rapidly reversible partial and complete membrane hemifusions (Melikyan and Chernomordik 1997; Kozlov et al. 2010) can take place at the inter-postsynaptic locations with increased lipid membrane turnover where GluA1 AMPA receptor subunit exocytosis is taking place. The localization of GluA1 AMPA subunits 25 nm away from the synaptic borders on the postsynaptic membranes (Jacob and Weinberg 2014) shows suitability for this mechanism.

## Locations of inter-postsynaptic LINKs in the visual cortex

One of the characteristic features of the locations of formation of inter-postsynaptic functional LINKs that induces internal sensations is that the lateral spread of activity through the inter-postsynaptic functional LINKs contributes to the oscillating potentials. In this regard, the observed oscillations that vary with function in the visual cortex (Fries et al. 2001; Gray et al. 1989; Beauchamp et al. 2012) indicate suitability for visual perception at this location. Inter-postsynaptic LINKs are expected to occur between the postsynapses on which the axonal terminals of the lateral geniculate nucleus (LGN) neurons synapse. LGN has two types of geniculo-cortical relay neurons to the visual cortex—type 1 neurons with large diameter (2–3.3 µm) axons and type 2 neurons with medium-sized (1–1.7 µm) axons (Ferster and LeVay 1978). They form synaptic connections with separate populations of cortical neurons. Type 1 axonal terminals arborize among a mixed population of simple and complex cells (pyramidal and spiny stellate cells) in layer IVαβ. The type 2 axonal terminals arborize among small spiny-dendrite stellate cells in layer IVc. Other studies have also made similar findings of synapse formation with the spiny apical dendrites of the visual cortical layer IVc (Peters and Feldman 1977; Wilson et al. 1976). Golgi staining studies following enucleation or light deprivation showed degeneration of axonal terminals that detected synaptic locations on different types of neurons such as the spiny stellate cells and spiny non-pyramidal cells of layer IV, pyramidal neurons of layers IV, V and VI, and the basal dendrites of layer III pyramidal neurons (Peters and Feldman 1977; Davis and Sterling 1979). The vertical extent of the visual cortical columns include neurons from all these layers (Hubel and Wiesel 1968) that match with the possibility of induction of semblances at the inter-LINKed postsynapses of preferably different neuronal types that belong to different neuronal orders. The formation of the internal sensation of perception concurrent with the arrival of sensory stimuli from the object requires certain modifications of the mechanism of semblance formation at the postsynapses to the LGN axonal terminals.

## Features associated with visual perception

**Fig. 2:**
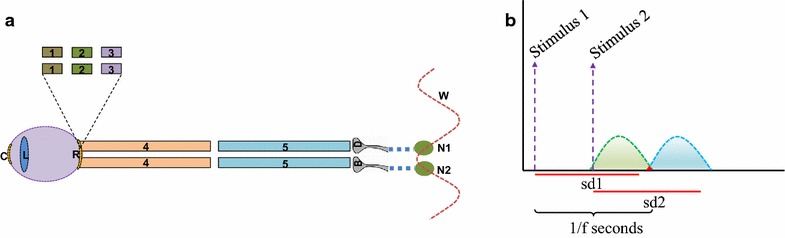
Diagrams showing total synaptic delay and merging of the frames of percepts. **a** In vivo perception time can be determined by the delay along the path of light inside the eyeball and the orders of neurons through which the stimulus travels to reach the neuronal orders in the visual cortex. Note that there is a minimum of five synaptic delay periods before the stimulus reaches the inter-postsynaptic functional LINK at the visual cortical neuronal orders. The five synapses will contribute to a delay of 5–10 ms. The lengths of nearly five cm each of the ganglion cell and the lateral geniculate neuronal (LGN) axons will add another 1 ms delay. The time required for the light to travel from the corneal surface to the pigment epithelium of the retina is taken as negligible. In summary, we expect a total delay ranging from 6–11 ms. *C* cornea; *L* lens; *R* retina [has layers *1*, *2*, *3* (shown in the inset) and the cell body of the layer *4*]; *1* pigment epithelium; *2* rods and cones; *3* bipolar cell; *4* ganglion cell axon (forms optic nerve); *5* lateral geniculate neuronal (LGN) axon. *B* and *D* Abutting postsynapses (dendritic spines) of the neurons in the visual cortex, where inter-postsynaptic functional LINK forms; *W* Oscillating potentials with its horizontal component contributed by the lateral spread of activity through the inter-postsynaptic functional LINK. *f* flicker fusion frequency. *N1* and *N2* are visual cortical neurons of the dendritic spines to which LGN terminals synapse. **b** A *graphical* representation showing temporally arriving stimuli above the flicker fusion frequency leading to the continuity in the percept. Stimulus 1 reaching the retina arrive at the neuronal location where the percept occurs after a delay sd which represents the synaptic delay. The units of perception continue to form and overlap (in *red triangle*) with the units of perception from the Stimulus 2 that arrives at the retina after a period of (1/flicker fusion frequency) seconds after the arrival of Stimulus 1. For the percept to become homogenous, the units of perception formed from the Stimulus 1 and Stimulus 2 should be capable of entangling with each other. A bidirectional overlapping symmetric process in the formation of units of perception is expected to allow maintaining homogeneity at the overlapping area (marked in *red*) between the percepts. *f* flicker fusion frequency, *sd* synaptic delay

There are five synapses from the retinal layer of rods and cones to the dendritic spines of the visual cortical neurons between which inter-postsynaptic functional LINKs are expected to take place. Due to the synaptic delay of 1–2 ms and conduction delay of 1 ms along the myelinated neuronal processes of the ganglion cell and the lateral geniculate neurons (an estimate based on their lengths), a total delay ranging from 6 to 11 ms is expected for the activity to propagate from the retina to reach the dendritic spines of the cortical neurons (Fig. 2a). When stimulation is delivered to the V1 layer of the striate cortex above the calcarine fissure, a visual percept of light is elicited in the lower part of the visual field and vice versa (Foerster 1931; Schmidt et al. 1996). The perceived phosphene lies within the visual hemi-field contra-lateral to the stimulated cortical hemisphere reflecting the retinotopic organization of the visual cortex (Tootell et al. 1988). Even though as per the third person observers, the activity from the visual stimuli arriving at the retina is inverted, the first-person internal sensation of percept is not inverted. This is possible when the retrograde “observing from within” path for the formation of percept of phosphene follows the path of arrival of visual sensory inputs to the visual cortex in the opposite direction.

## Merging of the frames of percept

Human observers perceive visual stimuli as continuous above the flicker fusion frequency (Herbst et al. 2013). Similarly, when looking at a lighted display, observers begin to notice a brief interruption of darkness if the latter is about 16 ms or longer (Watson 1986). It was shown experimentally that a minimum viewing time of 13 ms is required for visual comprehension (Potter et al. 2014). This indicates that when individual frames of visual stimuli are presented above a certain frequency, the percepts formed are entangled with each other to induce homogeneity of the percept (Fig. 2b). What neurobiological mechanism of perception can provide this entanglement? The possibility is that the mechanism of formation of internal sensations has a component that can function in an overlapping manner. How can the expected mechanism of formation of units of internal sensations of perception from a stimulus overlap with that of a second stimulus that arrives after a certain interval of time? This requires a process induced by the first stimulus that overlaps with that of a similar process induced by the second stimulus, such that continuity in the percept is maintained. In a system where activity along the inter-postsynaptic functional LINK contributes to the horizontal component required for the surface- or extracellular recorded oscillating potentials, units of perception get integrated to form the percept as a systems property.

## Columnar organization of the sensory cortices

Single unit analysis of the neurons in the somatosensory cortex shows groups of neurons organized in narrow vertically-oriented columns (Mountcastle 1957). The vertical arrays of neurons in the columns are more densely interconnected with each other than with neighboring neurons located laterally all around them. Further studies showed that neurons in primate visual cortical area 17 are preferentially driven by visual stimuli delivered to either eye and are selectively sensitive to short straight-line visual stimuli at limited angles of orientation (Hubel and Wiesel 1959, 1968, 1969). The horizontal diameter of the mini-columns in the neocortex ranges from 20 to 50 μm (Mountcastle 1997). Similar columnar organization was also observed in layer IV of the cortical columns of the barrel cortex using staining studies (Woolsey and Van der Loos 1970). Using voltage-sensitive imaging, the firing of several neuronal ensembles corresponding to cortical representations of orientations was demonstrated (Kenet et al. 2003). Calcium imaging studies at the cellular level of resolution have shown micro-architecture of the columnar organization in response to visual stimuli (Ohki et al. 2005). Even though orientation specificity of neuronal firing and columnar organization of visual cortex has been observed, the examination of neuronal firing alone could not provide any functional significance for the cortical columns (Horton and Adams 2005) when examined from a third-person frame of reference. Even though new model systems have been examined (Feldmeyer et al. 2013), a structure-to-function correlation remains unsolved. This becomes more important due to the redundancy among the sets of dendritic spines that can be activated to induce the same firing of a neuron (Vadakkan 2015b). Since evolutionarily conserved columnar patterns are expected to have certain functional attributes and, since perception is a first-person property, the functional significance of the cortical columns is yet to be discovered. The present work examined whether any feature at the neuronal input or output level controlled by the columnar organization can provide a mechanism for the first-person internal sensation of perception by modification of the units of internal sensation derived for memory (Vadakkan 2007; 2013) and whether its features can explain the entanglement of the frames of percepts.

The visual cortices process the fastest arriving visual sensory stimulus from an object that can evoke memories of the remaining features from the object that arrive slowly or may not reach it from a distance (for example, the sensations of smell, taste or touch). Among all the sensory stimuli, light has the maximum velocity. In this regard, for both the predator and the prey, the visual details are of paramount importance for their survival. Therefore, the structural features of the visual cortex are expected to be optimized to obtain the finest visual details of the object and the organization of cortical columns is likely to have a functional role. Similarly, the nocturnal life of rodents necessitates touch perception via whiskers, which can provide the finest surface details of objects so that the rodents can retrieve memories of the remaining qualities of the object. In this regard, the structural organization of the barrel cortex is expected to have a functional role. How can the columnar organization provide the finest details for the internal sensation of object perception?

## First-person inner sensation of perception

In contrast to the different higher brain functions, the internal sensation of perception occurs in real time at the arrival of the sensory stimuli. From the earlier sections, it can be seen that two essential features are associated with the formation of the internal sensation of perception. One is the need for an overlapping of the units of perception for achieving homogeneity of the percept above the flicker fusion frequency. The second is the limitations imposed by the functional perimeter of the cortical columns that necessitate the arrival of only a few input stimuli from the object at a given column. Since it was found that neurons within a column are selectively sensitive to straight-line visual stimuli at limited angles of orientation (Hubel and Wiesel 1959), visual stimuli from infinitesimally close locations from an object should be responsible for activating the neurons restricted to one column. Since several of these neurons within a column can exist in sub-threshold activated states, only a few synaptic potentials arriving at these neurons can lead to their firing. Therefore, to understand perception, a potential mechanism at the level of the postsynapses that is a modification of semblance formation is expected. Visual stimuli from infinitesimally close locations can reactivate existing inter-postsynaptic functional LINKs or induce rapidly reversible new inter-postsynaptic functional LINKs within each column. The potentials propagating through the inter-postsynaptic functional LINKs at the visual cortices are expected to provide the horizontal component (Vadakkan 2015b) of the oscillations of potentials taking place at these locations (Herculano-Houzel et al. 1999).

**Fig. 3:**
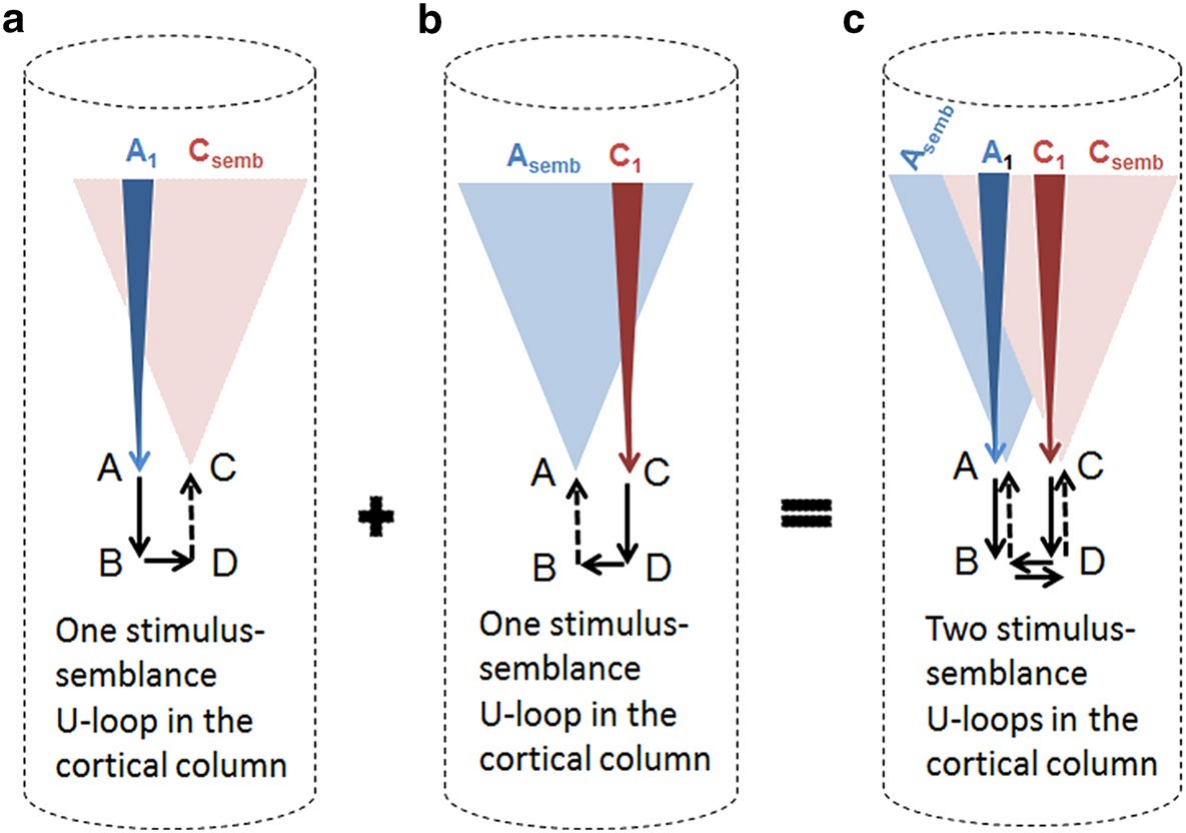
Diagram showing a symbolic representation of how the basic unit of the percept is formed within the cortical columns. Note that the lateral borders of the cortical columns enclosing the inter-postsynaptic functional LINK are represented by lateral walls of the *cylindrical shape*. This functional perimeter limits the number of inter-LINKed and inter-LINKable postsynapses at a given neuronal order. **a** Illustration showing the first stimulus activating sensory receptor set labeled *A*_1_ inducing the formation of the semblion marked C_semb_; **b** illustration showing the second stimulus activating the sensory receptor set labeled *C*_1_ inducing the formation of the semblion marked *A*_semb_; **c** the two sensory stimuli originate from infinitesimally close locations on the object. The simultaneous arrival of stimuli at the sensory receptors *A*_1_ and *C*_1_ can activate the inter-postsynaptic functional LINK from both sides inducing semblions at both inter-LINKed postsynapses *B* and *D*. The integral of these two semblions can form the basic unit of the percept called percepton

The columnar nature of the visual cortex restricts the number of islets of inter-LINKed postsynapses within them (Fig. 3a) and highly increases the probability that activity from stimuli arriving from infinitesimally close locations from an object will either (a) reactivate existing inter-postsynaptic functional LINKs from both its sides or (b) inter-LINK two abutted postsynaptic terminals by simultaneously activating them to induce semblances at the opposite postsynapses and reverse back rapidly. Such a mechanism is essential to explain the homogeneity of percept of the visual stimuli arriving above the flicker fusion frequency. Since inter-postsynaptic functional LINKs can be activated by stimuli from either side to evoke a semblance at the opposite inter-LINKed postsynapse, a bidirectional activation of the inter-postsynaptic functional LINK inducing the formation of units of perception is an ideal mechanism.

**Fig. 4:**
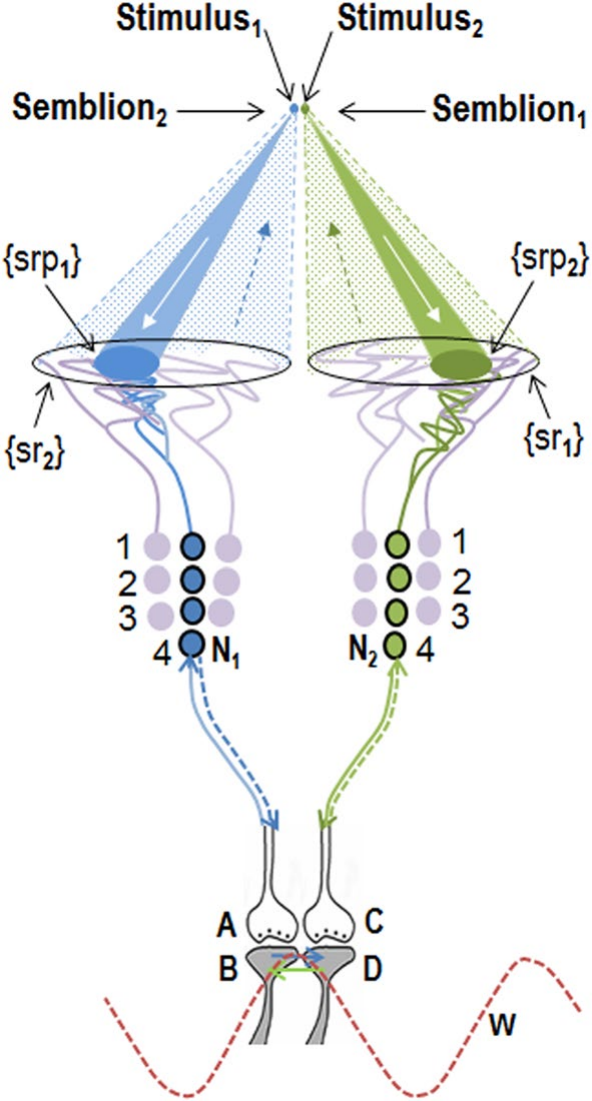
Diagram showing a mechanism for the formation of basic unit of perception. Stimulus_1_ and Stimulus_2_ arriving from infinitesimally close locations from an object simultaneously activate the inter-postsynaptic functional LINK from the opposite sides resulting in the simultaneous formation of Semblion_1_ and Semblion_2_ respectively. Stimulus_1_ activates the sensory receptor set {srp_1_} and this activity results in the activation of neuron N_1_ resulting in activation of the synapse *A*–*B*. This results in the activation of the inter-postsynaptic functional LINK *B*–*D* evoking a unit of semblance labeled Semblion_1_ from the receptor set {sr_1_}. Similarly, Stimulus_2_ activates the sensory receptor set {srp_2_} and activity results in activation of the neuron N_2_ resulting in the activation of the synapse *C*–*D*. This results in the activation of the inter-postsynaptic functional LINK B-D evoking a unit of semblance labeled Semblion_2_ from the receptor set {sr_2_}. Note that the semblance induced by one stimulus matches the other stimulus. This simultaneous formation of overlapping stimulus-semblance U-loops in opposite directions across the inter-postsynaptic functional LINK B-D is hypothesized to form the basic units of perception. Interruption of the formation of stimulus-semblance U-loops due to lack of sensory stimulus beyond the edges breaks the continuity in the formation of percept from the surface of the object. Simultaneous formation of stimulus-semblion U-loops arriving from a different plane provides percept of the scenery beyond the edges of the object. The neuronal paths through which stimulus reach the inter-postsynaptic functional LINK is given in blue and green. The neuronal paths through which extrapolation from the postsynapses up to the sensory receptors is carried out to estimate the semblance also include neurons and sensory receptors that are given in violet color. The directions of the stimuli and semblances are opposite to each other. Note that for simplicity, the semblions are drawn to a single point in contrast to their expected spanning over several sensory receptors as in the original derivation (Vadakkan 2013) and shown in Fig. 3 above. The overlapping of perceptons with that of the origin of sensory stimuli from the object occurs only for a limited distance from the eye. *w* Waveform in front of the inter-postsynaptic junction represents the oscillating surface- or extracellular-recorded potentials whose horizontal component has contributions from the spread of activity through the inter-postsynaptic functional LINK *B*–*D*

The stimulus and its corresponding semblance occurring at each side of an inter-postsynaptic functional LINK result in a two-way process of overlapping stimulus-semblance U-loops in opposite directions (Fig. 3b). The net semblance of two semblances formed by the sensory stimulus-semblance U-loops at one inter-postsynaptic functional LINK is called “percepton,” which is the basic unit of the percept (Fig. 4). Since perceptons are virtual packets of sensory stimuli that can activate a receptor subset (please refer to the derivation of semblance in Vadakkan 2013), they do not have any orientation, similar to that of the pixels in a digital image. It is the relative positioning and distribution density of the perceptons that gives rise to the quality of the final percept. Since lateral borders of the columns limit the number of islets of inter-postsynaptic functional LINKs within them, one possibility is that the integral of the perceptons from each column may have no orientation. Computational studies may help understand the lowest level at which possible pixilation effect takes place.

Since each postsynapse has the freedom to inter-LINK with multiple postsynapses, units of internal sensations are expected to overlap to induce a homogenous percept from a stationary object at a perceivable distance. The bidirectional flow of potentials through the inter-postsynaptic functional LINK also supports the observation of multi-directionality of the oscillating potentials recorded either extracellularly or extracranially. At the inter-postsynaptic functional LINKs, the integral of the perceptons form the percept irrespective of the location of the neuronal layer at which the inter-postsynaptic functional LINKs are formed at the synapses of the axonal terminals of the LGN neurons. Since the stimulus-semblion U-loops can be formed at every visual cortical column, similar percepts can be formed irrespective of the locations in the retina or inter-postsynaptic LINKs in the visual cortex that are activated by visual stimuli from infinitesimally close locations from the object.

In examining both the somatosensory and visual cortices, it can be seen that the vertical extent of the cortical columns includes neurons from almost all the horizontal layers except layer I (Mountcastle 1957; Hubel and Wiesel 1968). This indicates the possibility for increased interaction between the neuronal processes in layer I in the horizontal plane. The apical dendritic tufts from all the neuronal orders are anchored to the sub-pial region in layer I. Based on the observation that average inter-dendritic spine distance is greater than the average spine head circumference in pyramidal neurons (Konur et al. 2003) and astrocytic processes surround only 57 ± 11 % of the synapses where they occupied only less than half of the synaptic interface (Ventura and Harris 1999), the probability that dendritic spines of different neurons will interact with each other is very high. Based on the present work, the formation of different types of inter-postsynaptic functional LINKs (Vadakkan 2015a, b) can explain the nature of these interactions.

**Fig. 5:**
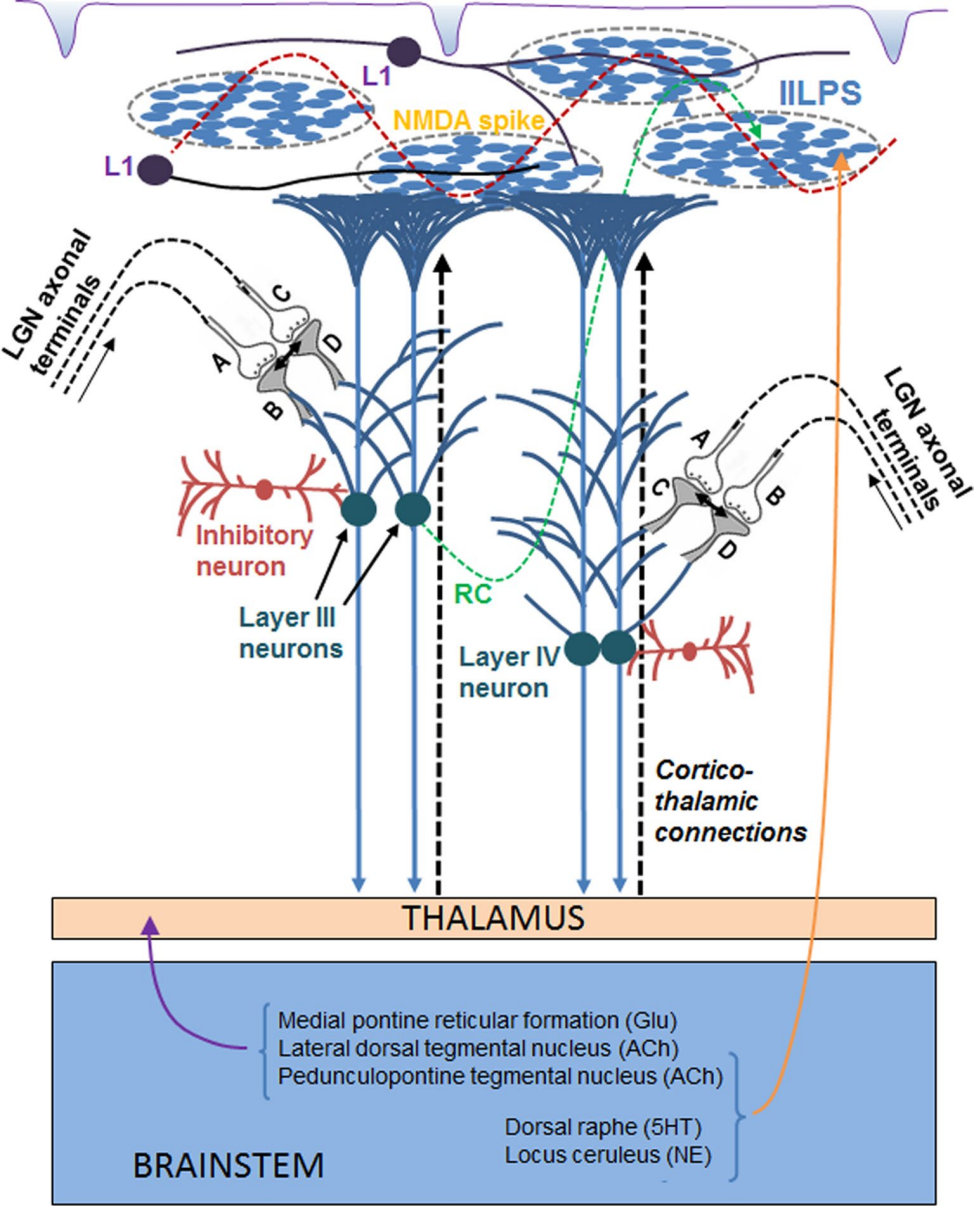
Diagram showing relative locations of formation of percept and non-specific semblances for C-semblance within a cortical column. Cortical columns do not include the neuronal layer I, one of the locations where NMDA spikes occur. Inter-postsynaptic functional LINKs expected to be responsible for NMDA spikes can contribute to the *horizontal* component for the surface-recorded potentials. Axonal terminals from lateral geniculate neurons (LGNs) synapse to the dendritic spines of the neurons of the remaining cortical neuronal orders. These synapses within the horizontally restricted volume of a column increase the possibility for visual stimuli from two infinitesimally close locations from an object to arrive at both ends of the inter-postsynaptic functional LINK to form overlapping stimulus-semblance U-loops in opposite directions and induce perceptons. Potentials from the inter-LINKed postsynapses can fire their neurons if the neurons are kept at subthreshold activated state short of very minimal voltage. Induction of percepton is a systems property of systems where lateral spread of activity through the inter-postsynaptic functional LINK contribute to the horizontal component and synaptic spread of activity contribute to the vertical component of oscillating potentials. The recurrent collaterals from different neuronal orders to the lower neuronal orders can contribute to the horizontal component of oscillation surface-recorded potentials. *RC* recurrent collateral, *L1* layer 1 cortical neuron. *A* and *C* Presynaptic terminals of the LGNs. *B* and *D* Postsynaptic terminals of the pyramidal neurons of layers III and IV. IILPS: Islet of inter-LINKed postsynapses. Glu: glutamate, Ach: acetyl choline, 5HT: 5-hydroxy tryptamine (serotonin), NE: nor-epinephrine (Figure modified from Vadakkan 2015a)

The lateral spread of activity along the inter-postsynaptic functional LINKS in layer I can contribute to both the horizontal component of the surface-recorded potentials and a large number of non-specific semblances contributing to the C-semblance for consciousness (Vadakkan 2010). In this context, recent evidence for the in vivo presence of dendritic NMDA spikes (Palmer et al. 2012; Cichon and Gan 2015; Sheffield and Dombeck 2015), which are synchronous activations of 10 to 50 neighboring glutamatergic synapses (Antic et al. 2010), provides an example for the spread of potentials between the spines at rest. The oscillating potentials also lead to both oscillating firing and sub-threshold activation of several higher order neurons. The axonal terminals of LGN neurons synapse with the dendritic spines of different types of neurons of all the cortical layers except that of layer I and possibly some portions of layer II. Along with the induction of perceptons at the inter-postsynaptic functional LINKs at different cortical layers, potentials from inter-LINKed postsynapses can lead to the firing of some of the sub-threshold activated neurons that are maintained by the baseline oscillating potentials (Fig. 5). If some of these neurons are upper motor neurons in layer V, it can lead to motor activity. This framework can accommodate (a) changes in the frequency of surface- or extracellular-recorded oscillations during eye opening or closure, (b) conscious visual perception, and (c) mechanism for visual perception in the absence of awareness, and coma vigil, a condition where the eyeballs of a comatose patient track a moving object as a reflex action.

In the context of the present work, it can be seen that the columnar organization brings spatial restriction only at the level of the inputs from the LGN neurons to the abutting postsynaptic terminals at different neuronal layers. This is essential for bringing sensory inputs arriving from infinitesimally close locations from an object to activate an inter-postsynaptic functional LINK from both its sides and induce perceptons. The induction of perceptons from the cortical columns is also required for bringing an effective pixilation effect for the percept (discussed below). Since the lateral spread of activity through the inter-postsynaptic functional LINKs contributes a horizontal component to the oscillating potentials for the systems function of percepton formation, horizontal connections between the columns are essential. Since the induction of perceptons takes place only at the level of the inputs from the LGN neurons to the abutting postsynaptic terminals at different neuronal layers, it is not affected by several non-matching findings detected when neuronal firing is given a central role in sensory perception (Boucsein et al. 2011). The induction of perceptons induces spontaneous firing of subthreshold-activated cortical neurons, which is supported by recent findings (Hagihara et al. 2015).

## Supporting features

There are several findings that can explain, support and supplement the present framework. Stringent criteria were kept in examining them so that they can provide sufficient mechanistic explanations for replicating the mechanism of perception in engineered systems. These include mechanisms for the homogeneity of percepts, perception of objects at apparent locations than the actual ones, explanation for the object borders, an explanation of purpose for columnar organization of the visual cortex, mechanisms taking place at a single location within the visual cortex for continued perception of changing scenarios and the minimum conditions, pathways involved in eliciting visual perception, using the example of pressure phosphenes and the lack of ability to perceive single photons.

### Entanglement of the frames of percepts

The arrival of the stimuli above the flicker fusion frequency results in homogeneity of the percept. This property was used to derive the mechanism of formation of perceptons (see the previous section “Overlapping of the frame of percept” and Fig. 2b). The formation of overlapping stimulus-semblance U-loops in opposite directions along the inter-postsynaptic functional LINK and the formation of islets of these LINKs within the cortical columns provide sufficient provision for achieving homogeneity of percepts induced above certain frequencies for both still and moving objects.

### Perception of objects at apparent locations

**Fig. 6:**
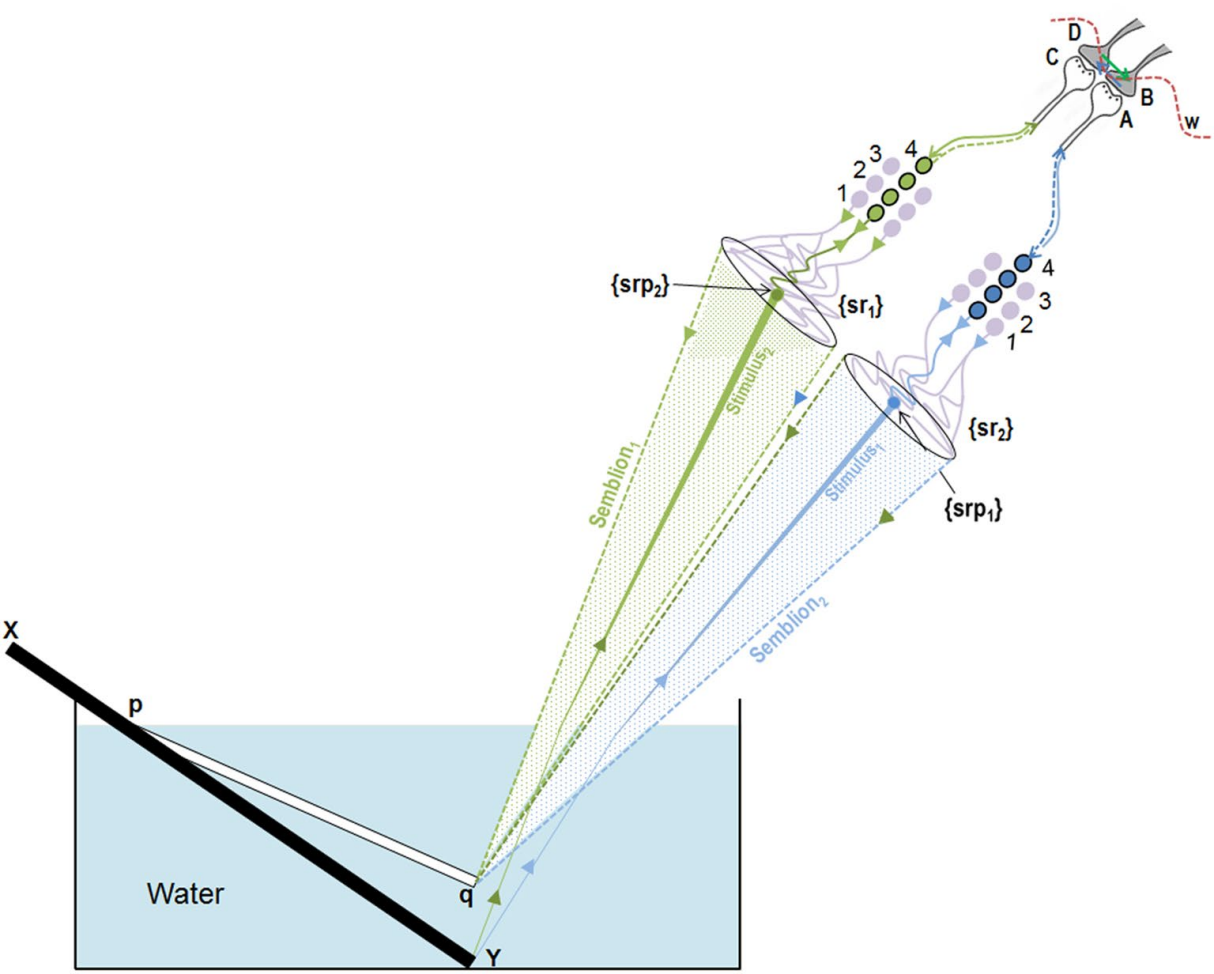
Diagram showing how the location of the percept can be different from the location of origin of the sensory stimulus. This is demonstrated using the experiment of refraction of light at the water–air interphase. Please rotate this page anticlockwise to view the formation of perceptons (modified from Fig. 4). XY is a rod placed in water. The perceived bent segment of the rod is shown as pq. Based on explanations in optics, the incident ray of light (afferent path) bends at the junction between water and air. However, there are no explanations how the efferent path of perception of the rod at pq occurs. Based on the present work, the perceptons (net semblions from both sides of an inter-postsynaptic functional LINK) get projected in straight lines outside the nervous system (Note that this is a description of the quality of the virtual internal sensation). The directions of the stimuli and semblances are opposite to each other. Note that for simplicity, the semblions are drawn to a single point in contrast to their expected spanning over several sensory receptors as in the original derivation (Vadakkan 2013) and shown in Figs. 3, 4 above. {srp_1_}: Sensory receptor set1; {srp_2_}: Sensory receptor set2; *A*–*B* and *C*–*D* synapses; *B*–*D* inter-postsynaptic functional LINK; {sr_1_}: sensory receptor set through which semblion1 is induced. {sr_2_}: sensory receptor set through which semblion2 is induced. 1,2,3,4: Neuronal orders. *W* oscillating potential that has horizontal component contributed by the lateral spread of activity through the inter-postsynaptic functional LINK *B*–*D*

It is known that the perception of an object at a location different from the original one occurs due to the refraction of light at the interface between the two different mediums. Even though an afferent path of light from the object towards the eyeball undergoes bending due to refraction, it is not known how the efferent path that determines the location at which the object is perceived occurs at a straight line from the eye. From the experiments of refraction of light from an object in water, stimulus-semblance U-loops through an inter-postsynaptic functional LINK converge to form the percept at a point close to the eye, which is different from the actual location of the object (Fig. 6). Even though the semblance induced at the inter-postsynaptic LINK takes the route along the path of the neural activity in a reverse order (Vadakkan 2013, 2015b), its projection outside the nervous system is along converging straight lines that determine the virtual location of the percept. Another example is the perception of the moon being closer to the earth than its actual position. Perceptons are overlapping with each other and with the origin of the stimuli when perceiving a near object. However, for both far objects and objects that undergo refraction, the percept does not overlap with the source of the stimuli.

### Percept of the object borders

The formation of sensory stimulus-semblance U-loops is required for the continued formation of the surface features of the percept when observing an object. The arrival of sensory stimuli in pairs from infinitesimally close locations from the objects or scenes stops beyond the edges of the object. This breaks the formation of the stimulus-semblance U-loops in the visual cortex using the stimuli from the object. This leads to the start of a new set of stimulus-semblance U-loops in response to the stimuli arriving from a different surface beyond the object borders and the integration of their perceptons forms the percept of the background. This provides a sufficient mechanism for perceiving object borders using the present framework.

### Diameter of the columns and the pixilation effect

The process of assembling the perceptons to obtain the percept can be compared to the pixels in a digital image. This may provide a mechanism for obtaining fine details for the internal sensation of perception. Since these virtual packets of internal sensations have no orientations for themselves, it is possible that the integral of all the perceptons from an islet of inter-LINKed postsynapses or even from a column may not have any orientation. The net percept will depend on the relative positions and distribution density of these integrated perceptons similar to the arrangement of pixels in a digital image. The size of the islet of inter-LINKable postsynapses will determine the net perceptons formed at that level. For the basic units of the percept to achieve properties similar to that of the pixels in an image, it is necessary to minimize the complexity of the perceptons. For this, the number of islets of inter-LINKed postsynapses from which perceptons are integrated should be kept as minimal as possible. This can be achieved by compartmentalizing the vertical arrays of neuronal orders in the sensory cortices within the laterally-restricted columns. This can explain a possible functional role for the cortical columns.

On the other hand, large islets of inter-LINKed postsynapses can increase the efficiency to continuously perceive a moving object. Therefore, a balance between the diameter of the column and the size of the islets of inter-LINKable postsynaptic terminals in each column determines both the clarity of the percept and the allowable spatial and temporal frequency of changing stimuli that can be accommodated by the system for continuous perception of a moving object. In summary, efficiency to perceive objects will be dependent on the number, size and efficiency of the mechanism of different types of inter-postsynaptic functional LINKs and the diameter of the cortical columns.

### Continued perception of changing sceneries

**Fig. 7:**
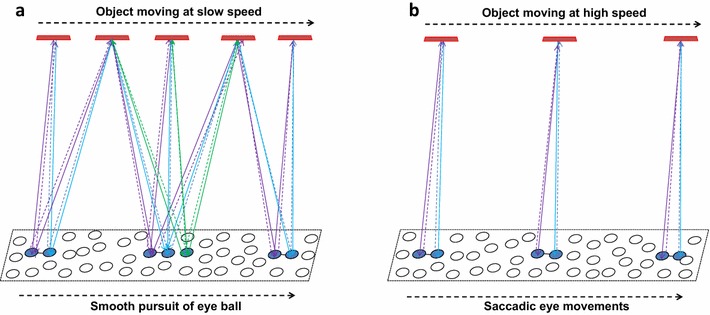
Schematic diagrams showing how the spatially distributed inter-postsynaptic functional LINKs enable perception of a moving object. **a** When the object moves at farther distances, the image falls on one end of the retina and moves along to the other end. This will lead to change in the locations where the activity reaches at the cortex, which is represented by a *rectangular shaped* area. The perceptons change their locations of formation as the object moves towards the *right side*. As the locations of formation of perceptons reach the end of the cortical field (represented by the *right side* of the *rectangle*), the smooth pursuit moves the eyeball slowly to the right side bringing the stimuli to arrive within the visual cortices. **b** When either the object moves very fast or the moving object is close to the eye, the perceptons formed when the inputs reactivate the inter-postsynaptic functional LINKs from its both sides become homogeneous by moving the eyeball quickly in a jumping (saccadic) manner

Based on the above descriptions, perceptons get integrated to form the percept as a systems property. This is explained for a stationary object. Adjacent loci on an object will be perceived by the induction of overlapping perceptons corresponding to these loci (Fig. 7). Rapid changes in visual stimuli require repeated formation of rapidly changing perceptons in one area of the cortex. This indicates that inter-postsynaptic functional LINKs responsible for inducing perceptons are most likely stable structures that are either innately determined or formed during the early days of perception and stabilized after a few events of perception or structures that are rapidly inducible, reversible and re-inducible. The perception of stimuli arriving from a moving object depends on the speed of the moving object and its distance from the eyes. Smooth pursuit by slow eyeball movement allows the perceptons to continue to form by the bidirectional activation of the same inter-postsynaptic functional LINKs. If the object moves faster than the formation of perceptons that can be overlapped, then it will lead to saccadic eyeball movements that will allow for continuity in percept formation. Any further increase in the velocity of the object will lead to head movement followed by body movement to maintain continuity in the formed percept. The above descriptions of the mechanism are based on the integral of semblances induced by spatial events of EPSPs. However, taking temporal events of EPSPs into account provides another mechanism for the entanglement of percepts and continued perception of moving objects.

### Perception of pressure phosphenes

The minimum required pathways and conditions for eliciting perception by a sensory system may be estimated by artificially activating the sensory path in the absence of a sensory stimulus. This can provide information about the nature and apparent location of percept formation in these conditions. Pressure phosphenes are transiently perceived as sensations of flashes of light when carried out in complete darkness by applying very gentle pressure on the lateral aspect of the closed eye, while looking towards the root of the nose (It should be carried out only by healthy individuals). The eyeball indentation leads to a non-uniform tangential stretch of the retina that exerts a locally variable depolarization of horizontal cells (Brindley 1955), providing the means to stimulate the afferent sensory pathways in the absence of light stimuli. It was later quantitatively analyzed (Grusser et al. 1989). The activity from the horizontal cell depolarization propagates towards the visual cortex. Phosphenes were also demonstrated by applying electrical current to the cortex using scalp electrodes and by trans-cranial magnetic stimulation (TMS) (Brindley and Lewin 1968; Kammer et al. 2001; Marg and Rudiak 1994; Merton and Morton 1980) and were found to involve the V1/V2 cortical areas (Cowey and Walsh 2000). The stimulation of different visual cortical areas by artificial means (Murphey et al. 2009) or by compression by mass-effect as in certain pathological conditions (Selimbeyoglu and Parvizi 2010) was also found to induce the internal sensation of visual percepts.

**Fig. 8:**
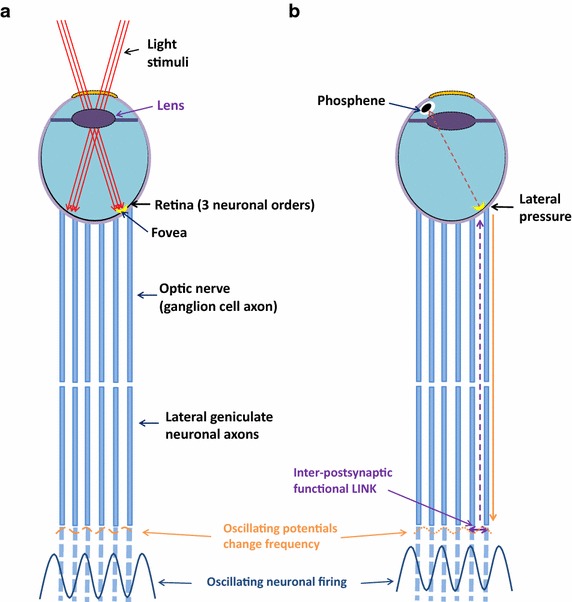
Diagrams of the right eye and its connections demonstrating pressure phosphene formation. **a** Right eye and its connections to the visual cortex viewed from above. It shows visual stimuli entering the eyeball and activating the retina. The activation then continues through the optic nerve towards the higher neuronal orders. For simplicity, crossing of the optic nerve fibers at the optic chiasm is not shown. At the synapses of the lateral geniculate nucleus (LGN) neurons with the cortical neurons, inter-postsynaptic functional LINKs are reactivated. Additional functional LINKs will be activated at higher neuronal orders for perception of more complex sensory features of the object. **b** View of the right eye from above, showing the formation of pressure phosphenes from stimulating the horizontal cells through lateral pressure over the eyeball. The procedure of application of gentle pressure over the eyeball is carried out in the *dark*. The phosphene is perceived when looking medially and upwards while applying gentle pressure over the lateral side of the eyeball. This activates a non-specific set of *horizontal cells*. As the activity pass through higher neuronal orders, a large number of inter-postsynaptic functional LINKs gets reactivated, activating the LINKed postsynapses and resulting in the formation of perceptons as a systems property. The net perceptons result in the internal sensation of the phosphene. The *broken arrow* shows the direction of the formation of the semblances. The oscillating patterns of neuronal activity is represented by the wave form

**Fig. 9:**
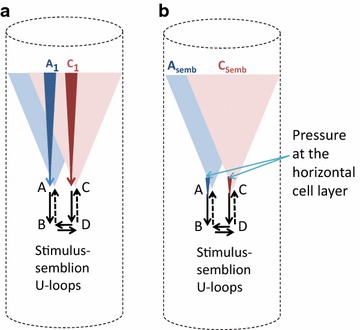
Diagrams comparing the perception of an object in the environment and that of pressure phosphene. **a** Diagram showing the simultaneous occurrence of input-semblance loops on both sides of the inter-postsynaptic functional LINK resulting in the perception of an object. *A*_1_ and *C*_1_ are sensory receptor sets activated by stimuli originating from infinitesimally close locations on the object [see Fig. 4 for details]; **b** Formation of the pressure phosphene percept from the application of pressure over the eyeball. According to the present work, the minimal requirement for the perception of pressure phosphenes is the simultaneous activation of inter-postsynaptic functional LINKs from its both sides that induce perceptons at the inter-LINKed postsynapses. Thus, simultaneous input-semblance U-loops inducing perceptons from both sides of the inter-postsynaptic functional LINK can be regarded as the basic structure–function mechanism required for perception of pressure phosphenes. A_Semb_: Semblance induced by the stimulus *C*_1_. C_Semb_: Semblance induced by the stimulus *A*_1_

Pressure phosphenes are perceived at the field of vision opposite to the location of application of pressure. This indicates that pressure phosphenes are perceived at a direction opposite to the direction of arrival of pressure stimuli over the posterior aspect of the eyeball similar to that of the normal visual perception. In this regard, the mechanism of percept formation of pressure phosphenes and normal visual perception has similarities in the direction at which percepts are formed. Since light-emitting cells were not observed in the retina (Bokkon 2009), how does the nervous system perceive light in its absence? Based on the present work, stimulating the retinal horizontal cells re-activates many inter-postsynaptic functional LINKs at the visual cortex (Fig. 8). This makes it possible to induce an operational mechanism producing the stimulus-semblion U-loops required for the internal sensation of the pressure phosphene (Fig. 9).

In patients with visual hallucinations due to the pathological accumulation of Lewy bodies in the inferior temporal cortex (Harding et al. 2002), it was shown that the neuro-psychiatric inventory score is positively correlated with the phosphene response rate and negatively correlated with the phosphene threshold (Taylor et al. 2011), indicating that the mechanisms of hallucinations and pressure phosphenes bear some relationship. In these patients, phosphenes induced by TMS are correlated strongly with the severity of visual hallucinations and not with stimulation intensity, indicating that the existing pathological process increases the number of operational units capable of inducing the perception of phosphenes. Moreover, increasing age was negatively associated with the phosphene response-rate, indicating that the number of operational units for visual perception decreases with age.

*Persistence of pressure phosphenes in the acquired blind:* Using an array of implanted electrodes, electrical stimulation delivered to the V1 layer of the visual cortex of the blind patients reproducibly resulted in a phosphene of a distant stationary star (Brindley and Lewin 1968). Phosphenes are only perceived by those blind individuals who have had some prior visual experience (Merabet et al. 2003). TMS-evoked phosphenes were also demonstrated in individuals with acquired blindness (Silvanto et al. 2007). A similar effect was also found through the magnetic stimulation of area V5/middle temporal (MT) in both hemispheres (Cowey and Walsh 2000). The demonstration of pressure phosphenes in acquired blindness indicates the possibility that inter-postsynaptic functional LINKs formed at the visual cortex during the period of active visual perception during the early years of life is likely maintained as long-lasting stabilized structural changes at the inter-postsynaptic membrane level.

*Perception depends on oscillating potentials:* In normal awake individuals, a scalp-recorded electroencephalogram (EEG) shows an alpha frequency background in the posterior occipital regions when the eyes are kept closed. These alpha waves change to beta frequency waves when the eyes are opened and light stimuli reach the visual cortex. This indicates that there is relative increase in the vertical trans-synaptic spread of activity between different neuronal orders compared to the increase in the horizontal component of oscillating potentials contributed by the reactivation inter-postsynaptic functional LINKs between the dendritic spines of the visual cortical neurons that synapse with the LGN axonal terminals. Extracellularly-recorded gamma oscillatory responses in the visual cortex were shown to be important in visual perception (Fries et al. 2001; Gray et al. 1989). The perception of phosphenes was shown to occur only if the artificial stimulation of the brain evoked high-frequency gamma oscillations in the temporo-parietal junction, a brain region associated with conscious perception of vision (Beauchamp et al. 2012). These findings indicate a possible relationship between the optimum frequency of surface- or extracellular-recorded oscillations and the optimal induction and integration of the perceptons for visual perception.

### Lack of ability to perceive single photons

Rods in the retina can respond to a single photon by hyperpolarization of their membranes during scotopic vision as observed by electrophysiological measurements (Baylor et al. 1979). A previous experiment has found that conscious perception of light can occur when at least 5 to 14 photons arrive at a population of 500 rods during dim-light vision (Hecht et al. 1942). Based on the present work, it is possible that more than one photon is required to increase the probability for activating at least the dendritic spines on two sides of one inter-postsynaptic functional LINK for one percepton formation, assuming that one percepton is sufficient to form a percept.

## Conservation of the mechanism

An ecosystem maintains a balanced relationship between the existence of the prey and the predator. When prey animals can survive beyond the age of reproduction, then their species survives. Since light travels faster than all other stimuli, visual sensations are perceived before all other sensations. By associating visual stimuli from a predator with other sensory stimuli such as those associated with being physically attacked, the animal can retrieve the memories of danger signals through the arrival of visual stimuli from the predator. This helps the animal escape from the predator quickly. On the other hand, a predator should be able to retrieve the memories of the sensation of food when visual sensations arrive from its prey. In the case of both the prey and the predator, the fastest arriving visual stimulus has to activate a very advanced mechanism to perceive clear visual details that can evoke memories of associatively learned slowly arriving stimuli from the remaining features. This likely explains the need for increasing the pixilation effect of perceptons for visual perception. Once such changes occur within the nervous system of the members of a species repetitively over many generations, conservation of these mechanisms is expected to occur.

The genetic code determines the division of embryonic cells and their differentiation, leading to the formation of neurons and the patterning of different neuronal types. There are proteins that guide neuronal migration, allowing a particular pattern of arborization of the dendritic tree. The dendritic spines of the sensory cortical pyramidal neurons of the same neuronal order are likely positioned close to each other and get abutted by the evolutionarily conserved cortical columns or barrels. It is possible that the closely abutted positioning of the postsynaptic terminals of different neurons may lead to the formation of inter-postsynaptic functional LINKs, providing a  horizontal component to the oscillatory potentials as the nervous system matures. As the premature newborns mature, newly positioned postsynapses at different neuronal orders lead to the continuous formation or activation of different inter-postsynaptic functional LINKs. This can explain the development of continuity in the tracings in the EEG (Selton et al. 2000) after the premature stage of development.

## Different sensory system in a different species

The inability to access first-person internal sensations makes it impossible to examine its formation using biological systems. One method to test the hypothesized mechanism is to look for the presence of comparable circuit features for perception of a different sensory stimulus in a different species. In this regard, well-studied olfaction in the fly *Drosophila* and its neuronal circuitry is a good candidate for examination. One of the key requirements is to find a mechanism for the first-person internal sensation of the perception of smell that is connected to third-person observed findings at the synaptic, neuronal and behavioral motor activity levels. Here, the fly nervous system is examined for a comparable circuitry.

## *Drosophila* olfactory nervous system

**Fig. 10:**
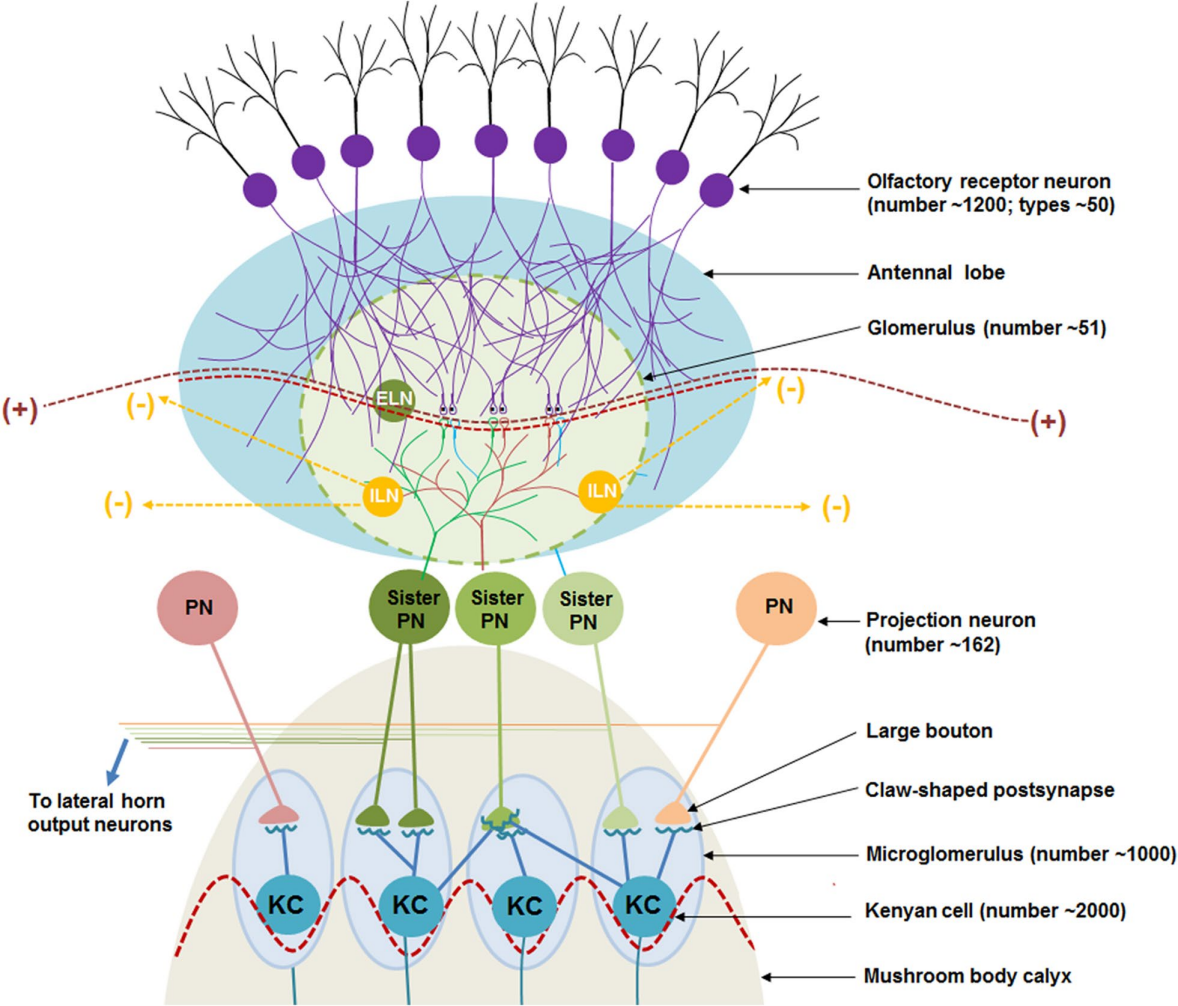
The anatomy of the Drosophila olfactory nervous system. The number of neurons or processes is given in the brackets. Nearly 1200 olfactory receptor neurons (ORNs) are of 50 different types based on the presence of odorant receptors. Their axonal terminals end in 50 different glomeruli. All of the ORNs that express one odorant receptor converge on to the same glomerulus in the antennal lobe. The presynaptic terminals from a rough average of 25 ORNs synapse with the postsynaptic terminals of nearly three sister projection neurons (PNs) within a single glomerulus. ORNs spike continuously at rest at the rate of an average eight spikes per second in the absence of ligands. Approximately three synchronous unitary ORN synaptic inputs drive one PN spike. In the absence of any odor, activity spread between the glomeruli through excitatory cholinergic local interneurons (ELN). Most odors cause ORN to fire at a rate of less than 50 spikes per second. Since all the ORNs that express a given odorant receptor converge onto the same glomerulus, one specific odorant is expected to activate PNs within one glomerulus. This leads to activation of inhibitory local interneurons (ILN) and  results in the inhibition of remaining glomeruli. PN axons innervate the mushroom body (MB) by terminating in large boutons and synapses with one claw each of many KCs. A single bouton connects to multiple Kenyan cell (KC) claws; but each claw synapses with only one PN bouton. PNs from different glomeruli synapse to the claws of the KCs by chance alone. The oscillatory potentials between the glomeruli is shown as a waveform through the ELNs

The neuronal orders and their connections in the olfactory nervous system of *Drosophila* are given below (Fig. 10). Each antenna contains nearly 1200 olfactory receptor neurons (ORNs) (Stocker et al. 1990). Each odorant receptor is expressed in multiple ORNs ranging from nearly 10 to 100 per antenna (Shanbhag et al. 1999). Based on the presence of odorant receptors, there are nearly 50 different types of ORNs (Wilson 2013). Most ORNs respond to multiple ligands and most individual ligand activates multiple ORNs (Wang et al. 1998; Malnic et al. 1999; Vosshall et al. 2000). In both vertebrates and insects the receptive range of each ORN is determined by the expression of specific functional members of a large family of odorant receptors (Buck and Axel 1991; Clyne et al. 1999). ORNs spike even in the absence of ligands (Wilson 2013) with an average firing rate of 8 spikes per second (de Bruyne et al. 2001). Most odor-evoked ORN firing rates are low (less than 50 spikes per second) compared to the maximum ORN firing rate of nearly 300 spikes per second (Hallem and Carlson 2006). All of the ORNs that express a given odorant receptor converge on to the same glomerulus (Malnic et al. 1999; Vassar et al. 1994 Gao et al. 2000 Mombaerts 2004; Ressler et al. 1994), a structure within the antennal lobe. Thus, signals from different odorant receptors are spatially segregated at the first synaptic relay in the brain. The ORN’s receptor specificity is linked to specific glomerular target in the antennal lobe (of insects) or olfactory bulb (of vertebrates) to which they project.

The antennal lobe has nearly 50 glomeruli (Hallem and Carlson 2004). Each glomerulus receives inputs from precisely the same set of ORNs and transmits information in nearly identical spike trains (Kazama and Wilson 2009) to downstream brain areas. For example, exposure to carbon dioxide (CO_2_) activates only one glomerulus (Suh et al. 2004). Approximately, three synchronous unitary ORN synaptic inputs are required to drive the neuron in the next neuronal order called a projection neuron (PN) from its resting potential to its spike initiation threshold (Gouwens and Wilson 2009). Most PNs have dense dendritic arborisations within the glomeruli. On average, 3 PNs (sister PNs) extend their dendrites inside a single glomerulus (Stocker et al. 1990; Marin et al. 2002; Wong et al. 2002; Schlief and Wilson 2007; Buonviso et al. 1991). Due to spontaneous spiking inputs arriving from the ORN, PNs also spike spontaneously. Olfactory perception in the fly is initiated by the binding of an odorant to the receptors on an ensemble of ORNs in the antennae, resulting in the activation of a specific topographically fixed combination of glomeruli (Ng et al. 2002; Wang et al. 2003). A given ORN targets its axonal terminals to one glomerulus and a given PN targets its dendritic terminals to only one glomerulus (Jefferis et al. 2005). There are populations of both excitatory and inhibitory local interneurons (ELNs and ILNs) with processes that span across multiple glomeruli with little glomerular specificity (Shang et al. 2007; Hong and Wilson 2015). Odor-evoked oscillatory synchronization of neurons is mediated by different local interneurons (Tanaka et al. 2009).

PN projects axons that bifurcate to innervate two distinct brain regions: the mushroom body (MB) and the lateral horn (LH). PN axons innervate the MB by terminating in large boutons (Marin et al. 2002; Wong et al. 2002). A single bouton connects to multiple Kenyan cell (KC) claws to form a discrete anatomic structure called the micro-glomerulus (Yasuyama et al. 2002; Leiss et al. 2009; Butcher et al. 2012). Each KC typically has five to seven claws and each claw synapses with only one PN bouton (Leiss et al. 2009); however, later estimations show that each KC contacts PN boutons at 18–24 PN presynaptic sites (Butcher et al. 2012). Electrophysiological and optical imaging studies show that odorants activate subpopulations of KCs distributed across the MB without any spatial preference. The classification of either glomeruli or KCs on the basis of several shared anatomic and functional features failed to show a structured input onto individual KCs (Caron et al. 2013). The emerging model for the functions of the MB is that each KC receives inputs from a combination of glomeruli randomly chosen from the non-uniformly distributed glomerular projections to the MB (Caron et al. 2013). Odorant-evoked local field potential (LFP) oscillations are seen in the MB (Paulk et al. 2013).

Examination of the neuronal activity and electrophysiological findings of the fly olfactory system from a third-person frame of reference have led to several questions (Table 1) that remain unsolved (Wilson 2013). Since the olfactory perception is a first-person property, examination from a first-person frame of reference is likely to find the solution to these questions.

**Table 1:** Questions that were emerged from observations made from a third-person view and require answers to explain their functional attributes towards the first-person property of perception

1.	Why are the ORNs active spontaneously?
2.	Why is this not making a fundamental limit on the ability of downstream neurons to detect transient odor stimuli?
3.	What mechanism causes specific percepts to be formed?
4.	What are the functions of ELNs?
5.	Why are PN odor responses so sensitive to weak inputs and why are they so reliable?
6.	Why would it be useful to segregate each ORN type into a different glomerulus?
7.	What dictates the innate hedonic valence of a particular pattern of PN activity?
8.	What type of computations take place in the glomeruli?
9.	Why do neurons in KC cells oscillate?
10.	How does the fly enjoy good percepts?

## Olfaction in the fly

Based on the derivation of perception in the visual system, the following observation can be made in the olfactory system of the fly. Here, third-person findings in the neural circuitry of the fly are translated to the first-person descriptions of the mechanism of internal sensation of olfactory perception based on the derived mechanism of visual percept formation. All the ORNs that express the same receptor type converge to the same glomerulus. Each PN neuron, which is the second-order neuron, receives direct input from just one single ORN type. The baseline firing frequency of eight spikes per second of ORN neurons does not induce any perception of smell. Olfactory perception in the fly is initiated by the binding of odorant molecules to the olfactory receptors on a group of ORNs in the antennae, resulting in the activation of a unique and topographically fixed combination of glomeruli in the antennal lobe, depending on the types of odorant receptors that the odorant molecules can activate.

**Fig. 11:**
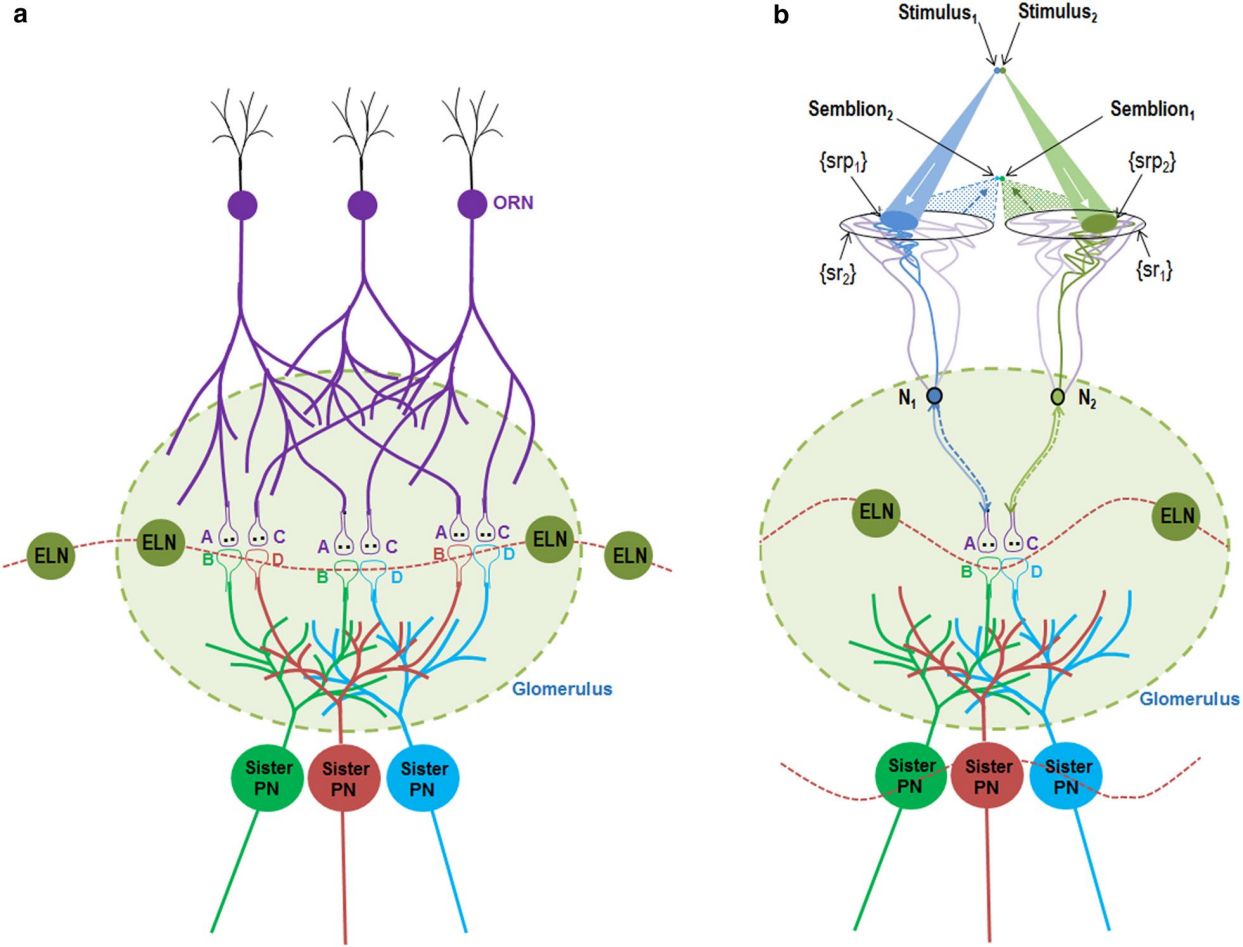
Schematic diagram showing the mechanism of olfactory percept formation within a glomerulus. **a** Spread of activity through the neuronal processes in the absence of odorants. The baseline firing of the olfactory receptor neurons (ORNs), lead to spread of activity to the synapses between the ORNs and the projection neurons (PNs). Spread of activity through the excitatory local neurons (ELNs) from one glomerulus to other glomeruli results in oscillating activity across different glomeruli in the antennal lobe. Two postsynapses each from the corresponding three different sister PNs whose dendrites are located within a single glomerulus are shown. Based on the present work existing inter-postsynaptic LINKs within each of the different glomeruli can contribute to horizontal component that can trigger oscillations of potentials among the glomeruli. The integral of all the non-specific semblances induced at the inter-postsynaptic LINKs is called C-semblance that can contribute to the fly attention. *A* and *C* are the presynaptic terminals of the ORNs. *B* and *D* are the postsynaptic terminals of two different PNs within a glomerulus. **b** Induction of perceptons in the presence of an odorant. Two synapses between two ORNs and two sister PNs within the glomerulus along with their inter-postsynaptic LINK *B*–*D* is shown. In the context of background C-semblance, the stimulus-semblion U-loops form at the inter-postsynaptic LINK *B*–*D* to induce percepton (as explained in Fig. 4). Note that the semblions are shown to overlap closer to the olfactory receptors than the actual source of the odorant. This enables localization of the odor close to the olfactory receptors, in contrast to the visual perception. The entanglement of perceptons provides the conformation for the percept of a specific smell. Percept of a specific attractive smell formed within a glomerulus can trigger motor actions to the fly along the concentration gradient as a response to increasing percepts, the fly can reach towards the source of food. Note that the oscillating potential wave form that extended beyond the single glomerulus in the absence of odorants got limited to that glomerulus alone due to the spread of inhibitory activity to the other glomeruli through the inhibitory local neurons (ILNs) during perception

All the ORNs expressing the same odorant receptor wire precisely to the same PNs. All the terminals of the dendritic arbor of one PN reach one glomerulus and mix with the dendritic arbors of sister PNs. Stimulation of a specific type of ORN receptor by a single type of ligand activates corresponding postsynaptic terminals of the PNs within a glomerulus. Restricting the arrival of activity to one glomerulus will increase the probability for them to reactivate the inter-postsynaptic functional LINKs from their both sides, which will lead to the generation of stimulus-semblion U-loops to form the perceptons. Entanglement of the perceptons results in the homogeneity of the percept of smell. The net semblance of all the perceptons forms the percept of a specific smell (Fig. 11). This is expected to occur with an odorant-induced ORN activation at a frequency nearly 50 spikes per second for most odors (Hallem and Carlson 2006). The requirement of this frequency of ORN spikes for a continuous perception of smell is similar to the 60 Hz frame-rate of visual stimuli required for visual perception of a movie. The induction of semblances at the inter-postsynaptic functional LINKs in the glomerulus agrees with the previous assessment that the glomeruli are discrete processing channels (Wilson 2007).

By segregating each ORN type into a different glomerulus, the number of stimulus-semblion U-loops formed in response to a specific odor can be increased for increasing the number of perceptons formed. While one glomerulus is activated, inhibitory local interneurons (ILN) inhibit all the remaining glomeruli (Hong and Wilson 2015), enabling the specificity of the percept for that smell. It is explained that the odor sensitivity of the PN is attributed to the background lateral spread of excitation (Wilson et al. 2004). The restriction of stimulus-semblion U-loops of percept formation occurring only in one glomerulus (and inhibited in the other glomeruli) may explain why the odor responses of PN are sensitive to weak inputs (Bhandawat et al. 2007). The perceptons formed at the inter-LINKed postsynapses of a glomerulus are expected to induce oscillatory synchronization of spikes in groups of PNs in response to odor stimulation (Laurent 1999). The odor-induced ORN-PN synaptic activation can contribute to the vertical component and the spread of potentials through the inter-postsynaptic functional LINKs between the PN neurons contribute to the horizontal component of the oscillating potentials, making oscillatory synchronization of spiking in groups of PNs necessary for fine odor perception (Stopfer et al. 1997).

The reactivation of a maximum number of inter-postsynaptic functional LINKs evokes the peak quality of percept for smell. A given odor is not expected to activate non-specific receptors even at the highest concentrations. It is observed that many odors are attractive at low concentrations but aversive at higher concentrations (Wang et al. 2001; Schlief and Wilson 2007). Since the number of the inter-postsynaptic functional LINKs and the speed of the spread of depolarization through them are limited, an increased firing rate of ORNs in response to increased concentration of an odor may not allow stimulus-semblion U-loops to operate efficiently, possibly at the stage of entanglement of perceptons or by altering the frequency of oscillating potentials within the glomerulus.

 Since a large number of postsynapses is expected to be inter-LINKed by innate mechanisms that enable the fly to smell food and survive, the presence of islets of LINKed inter-postsynapses is anticipated at the time of birth. It may be noted that perceptons can be formed even if the inter-postsynaptic functional LINKs occur between the postsynapses that belong to the same PN. A comparable location in mammals is the dendritic excrescences on the dendritic tree of individual CA3 neurons in the hippocampus. The present circuit can provide a mechanism for inducing internal sensation of perception at the inter-LINKed postsynapses of the PNs at the ORN-PN synaptic junctions and its correlation with the spiking of GABAergic ILNs and possibly the latter’s phase-locking with the LFP oscillations in the MB (Tanaka et al. 2009).

## Relation between fly perception and attention

Oscillating potentials and the arrival of background sensory stimuli are expected to induce non-specific semblances and were explained to contribute to C-semblance for consciousness (Vadakkan 2010). Even though unconscious perception and motor action can occur, for the purpose for building this framework only conscious perception is considered. It is possible that the interaction between the perceptons for a particular sensation and the C-semblance determines whether the perception, retrieval of memories of previously learned items and response-motor action takes place consciously or not.

It is known that ORNs spike even in the absence of ligands (Wilson 2013) with an average firing rate of 8 spikes per second (de Bruyne et al. 2001). This spontaneous ORN firing activates dendritic spines of a large number of PNs in different glomeruli by the lateral spread of activity through the ELNs. Based on the concept of the induction of semblances, the baseline activation of postsynapses reactivates many inter-postsynaptic functional LINKs and activates their inter-LINKed postsynapses to induce non-specific semblances. The net semblance from all the semblances induced at the inter-postsynaptic functional LINKs in all the glomeruli that are interconnected through the ELNs is a non-specific C-semblance responsible for the formation of the fly's attention. The conformation of the C-semblance will depend on the frequency of oscillating potentials among the glomeruli. The lateral spread of activity through the ELNs and the inter-postsynaptic functional LINKs of the PNs can contribute to the horizontal component of the oscillating potentials between the glomeruli. The background oscillating potentials giving rise to attention can be considered a background systems property necessary for the induction of olfactory perception.

## Glomeruli are comparable to the cortical columns

 Based on the present work, limiting the number of inter-LINKable postsynapses is essential to provide visual perception properties similar to that of the pixilation in a digital image. This is achieved by limiting the lateral borders of the possible expansion of the islets of inter-postsynaptic functional LINKs by the vertically arranged cortical columns. It is described in a previous section that by increasing the number of cortical columns, the pixilation effect by the perceptons is increased. However, the number of different types of smell percepts induced is expected to be much smaller than the number of visual percepts. This is evident from the presence of only 50 different types of ORNs (Wilson 2013) among the 1200 ORNs in each antenna (Stocker et al. 1990). All of the ORNs that express a given odorant receptor converge onto the same glomerulus (Malnic et al. 1999; Vassar et al. 1994; Gao et al. 2000; Mombaerts 2004). The antennal lobe has nearly 50 glomeruli (Hallem and Carlson 2004), which is same as the number of ORN types. On average, 3 sister PNs extend their dense dendritic arbor inside a single glomerulus (Stocker et al. 1990; Marin et al. 2002; Wong et al. 2002). The spatial limitation of the dendritic spines of the sister PNs within a glomerulus ensures that only one type of olfactory receptor activation reaches one glomerulus. Therefore, the induced stimulus-semblion U-loops will provide similar perceptons within a glomerulus. In this regard, the spatial restriction of dendritic arbors of only three PNs within a single glomerulus is similar to a cortical column in the visual cortex. The induction of perceptons by the activation of the inter-postsynaptic LINKs of the dendritic spines of mitral cells in vertebrates is expected to be similar to that occurring at the dendritic spines of the PNs in insects.

## Testable predictions from the mechanism

1. Based on the present work, innately determined and pre-existing inter-postsynaptic functional LINKs form the major mechanism responsible for percept formation. The pre-existing ones are expected to have inter-postsynaptic membrane hemifusions likely stabilized by trans-membrane proteins. Evidence for these can be examined in the visual cortex and olfactory glomerulus by using advanced high-resolution microscopes.
2. The second possible mechanism of percept formation by rapid removal of hydration repulsion at the inter-postsynaptic locations and its rapid reversal by rehydration can be examined in the visual cortex and olfactory glomerulus. This is likely to require developing novel techniques.
3. By changing the frequency of oscillating potentials in the glomerulus, the net semblance for olfactory perception can be changed. This will alter olfactory perception.
4. Blocking large number of inter-postsynaptic functional LINKs either in the visual cortex or in the glomerulus is expected to alter the horizontal component of oscillating potentials, which will alter the frequency of oscillating potentials and disrupt visual or olfactory perception respectively.

## Conclusion

The present framework used third person observations to construct a feasible mechanism for the first-person internal sensation of perception and has explained a large number of findings made at different levels. In a state of background semblances induced by oscillating patterns of activation of several postsynapses that contributes to the internal sensation of awareness, the arrival of sensory stimuli induces perceptons that are basic units of perception. The lack of orientation for the perceptons indicates their likely mechanism of integration to form the percept is likely similar to that of pixels in a digital image. The present work has provided a new explanation for the spatial restriction of the cortical columns that can provide mechanisms for accommodating the requirements for the induction of perceptons, and incorporating a pixilation-like effect in the percepts. The presented framework has also provided answers to a large number of questions arising when the circuitry for perception is examined from a third-person frame of reference (Wilson 2013).

The percept of the smell of nutritious food that is deemed attractive is likely evolved from an evolutionary selection process due to the survival of flies that fed on those food items. Even though light travels much faster than smell, odorant molecules can reach the fly even from hidden locations from where visual inputs won’t reach the fly. Therefore, perceiving smell is much more efficient than visual stimuli for detecting the presence of food. Flying towards the direction of increasing stimulus gradient of odorants can lead the fly towards the source of food. The presence of a circuitry for the first-person visual perception in mammals and olfactory perception in the fly *Drosophila* supports the view that unitary elements for the formation of the internal sensation exist across different members of the animal species and across different sensations. As the complexity of the nervous system varies, the nature of the basic quality of percepts is likely to change. *Drosophila* genetics can be used to design experiments to perturb the functioning of the circuitry at different levels to understand the details of the mechanism. A similar mechanism is expected to operate for the perception of other sensations in the nervous system.

## Abbreviations

CO_2_: carbon dioxide
EEG: electroencephalogram
ELN: excitatory local interneuron
EM: electron microscopy
EPSP: excitatory postsynaptic potential
ILN: inhibitory local interneuron
IPSFL: inter-postsynaptic functional LINK, inter-postsynaptic LINK, inter-LINK
KC: Kenyan cell
LFP: local field potential
LH: lateral horn
LN: local interneuron
LGN: lateral geniculate nucleus
LINK: inter-postsynaptic functional link
MB: mushroom body
MBON: mushroom body output neuron or extrinsic output neurons
ms: milliseconds
MT: middle temporal
ORN: olfactory receptor neuron
PAM: proto-cerebral anterio-medial cluster
PN: projection neuron
Postsynapse: postsynaptic terminal or dendritic spine or spine or input terminal
Spine: dendritic spine or postsynapses or postsynaptic terminals or input terminal
TMS: trans-cranial magnetic stimulation
U-loops: overlapping stimulus-semblance U-loops in opposite directions
